# A Quality Improvement Project to Improve After-visit Summary Patient Instructions in a Pediatric Multidisciplinary Neuromuscular Program

**DOI:** 10.1097/pq9.0000000000000743

**Published:** 2024-07-10

**Authors:** Agathe M. de Pins, Dorothy Adu-Amankwah, Kristin A. Shadman, Skylar M. Hess, Cordelia R. Elaiho, Liam R. Butler, Sheena C. Ranade, Brijen J. Shah, Robert Fields, Elaine P. Lin

**Affiliations:** From the * Department of Pediatrics, Icahn School of Medicine at Mount Sinai, New York, N.Y.; † Department of Pediatrics, University of Wisconsin School of Medicine and Public Health, Madison, Wis.; ‡ Medical College of Wisconsin, Milwaukee, Wis.; § Department of Orthopedic Surgery, Icahn School of Medicine at Mount Sinai, New York, N.Y.; ¶ Henry D. Janowitz Division of Gastroenterology, Icahn School of Medicine at Mount Sinai, New York, N.Y.; ‖ Beth Israel Lahey Health; ** Division of General Pediatrics, Boston Children’s Hospital, Harvard Medical School, Boston, Mass.

## Abstract

**Introduction:**

Multidisciplinary clinics aim to coordinate care between multiple specialties for children with medical complexity yet may result in information overload for caregivers. The after-visit summary (AVS) patient instruction section offers a solution by summarizing visit details and recommendations. No known studies address patient instruction optimization and integration within a multidisciplinary clinic setting. This project aimed to improve the quality of patient instructions to support better postvisit communication between caregivers and providers in a multidisciplinary pediatric neuromuscular program.

**Methods:**

A multidisciplinary stakeholder team created a key driver diagram to improve postvisit communication between caregivers and providers in the clinic. The first specific aim was to achieve an 80% completion rate of AVS patient instructions within 6 months. To do so, a standardized electronic medical record “text shortcut” was created for consistent information in each patient’s instructions. Feedback on AVS from caregivers was obtained using the Family Experiences with Coordination of Care survey and open-ended interviews. This feedback informed the next specific aim: to reduce medical jargon within patient instructions by 25% over 3 months. Completion rates and jargon use were reviewed using control charts.

**Results:**

AVS patient instruction completion rates increased from a mean of 39.4%–85.0%. Provider education reduced mean jargon usage in patient instructions, from 8.2 to 3.9 jargon terms.

**Conclusions:**

Provider education and caregiver feedback helped improve patient communication by enhancing AVS compliance and diminishing medical jargon. Interventions to improve AVS patient instructions may enhance patient communication strategies for complex medical visits.

## INTRODUCTION

For children with complex health care needs, including cerebral palsy and neuromuscular disorders, multidisciplinary programs have emerged as a way to improve care coordination and provide more efficient care to families.^1–3^ Typically, families see multiple specialists in a centralized manner through these clinics. These clinics may improve outcomes such as decreased symptom burden, increased patient satisfaction, and reduced emergency room visits.^4–7^ Families must retain complex and varied information during these sessions, and optimal methods for sharing assessments and plans with families are not fully understood.^8,9^

The Center for Medicare and Medicaid Services recommends that physicians provide clinical summaries for each office visit.^10^ The after-visit summary (AVS) is a document given to patients after their encounter. It is expected to comprehensively present an overview of the encounter, medication list, care guidance, provider contact information, and future appointments.^11–13^ It is an important communication tool between providers, patients and caregivers. Yet, limited literature addresses maximizing its effectiveness in pediatric populations or the unique context of multidisciplinary clinics.^14–16^ Although the AVS has the potential to facilitate shared decision-making and effective communication, some studies have shown its design is not consistently optimized to convey valuable information clearly and understandably.^17^ One primary challenge in patients’ adoption and use of AVS patient instructions is the presence of medical jargon within providers’ documentation.^18,19^ Excessive use of medical terminology can potentially create misunderstandings regarding care plans, treatment, and medical conditions, ultimately leading to mistrust.^20^

The global aim of this project was to enhance postvisit communication between providers and caregivers in our institution’s multidisciplinary pediatric neuromuscular program. The specific aims were to achieve an AVS patient instructions completion rate of 80% within 6 months and reduce the use of medical jargon in AVS patient instructions by 25% within 3 months.

## METHODS

This project’s comprehensive pediatric neuromuscular care program at an urban pediatric tertiary care hospital started in August 2019. Patients are primarily referred from the institution’s outpatient complex care program. A typical clinic session averages five patients and nine providers from different specialties. Examples of patients referred to the program include children with spastic quadriplegic cerebral palsy and seizure disorders or children with muscular dystrophy requiring life-sustaining equipment. The program meets once or twice monthly and includes pediatric providers from orthopedics, neurology, pulmonology, nutrition, neurosurgery, urology, rehabilitation, dentistry, and primary care. Each patient sees 6–8 providers per visit and has an AVS attached for each provider encounter.

Each AVS containing the patient instructions can be printed for the patient after their encounter with a specific provider and is also available electronically on the patient portal. Patient instructions from each provider are specific to their specialty. Each patient receives multiple AVS, as each provider has their encounter linked to their individual AVS and patient instructions.

**Fig. 1.**
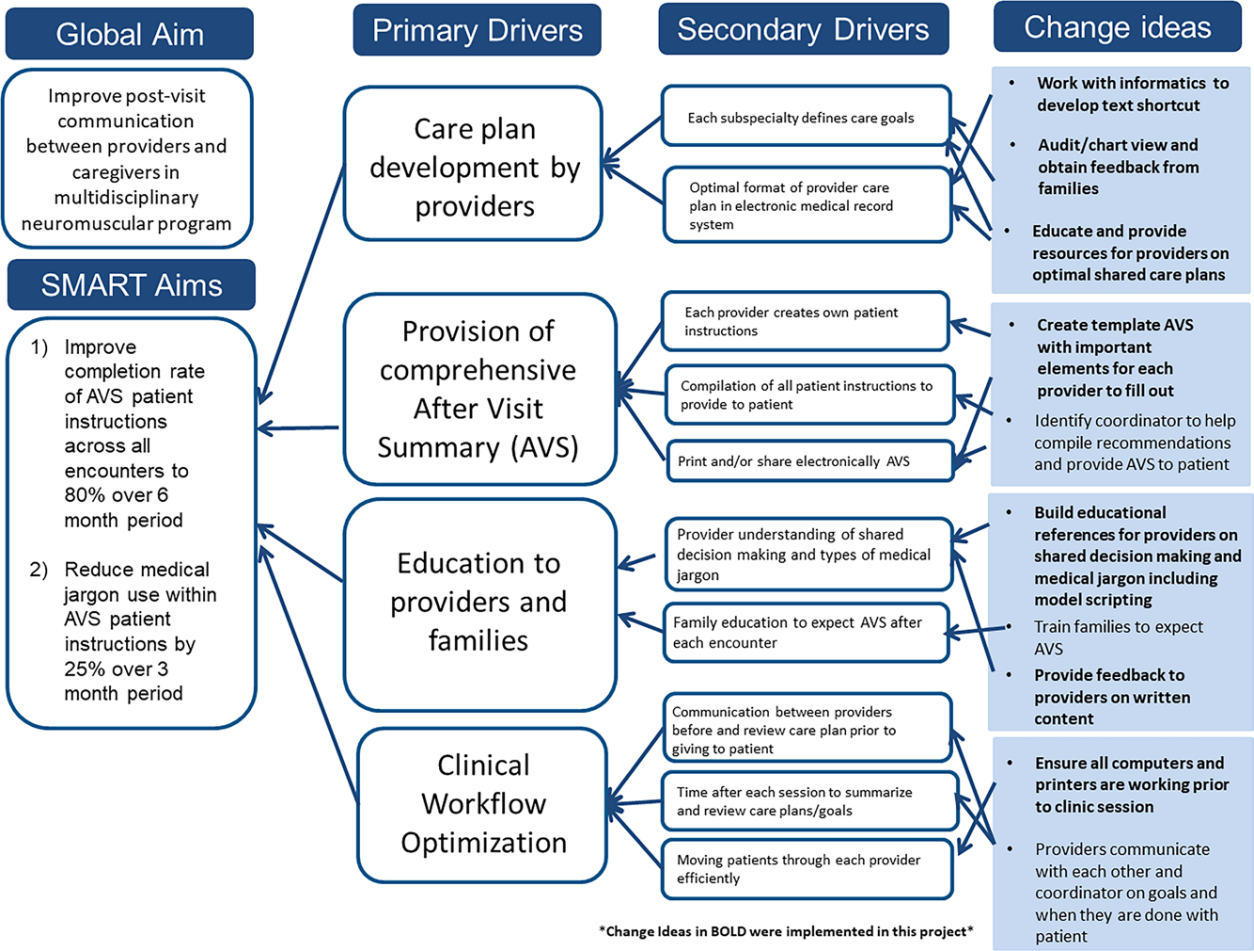
The key driver diagram presents the primary and secondary drivers and change ideas for improving postvisit communication between providers and caregivers in a pediatric multidisciplinary neuromuscular program.

In May 2021, a multidisciplinary stakeholder team composed of clinicians and care coordination support staff (nurses, social workers, care coordinators, and administrative staff) noted a large variation in completion and content between each AVS in the clinic. Using the Model for Improvement from associates in process improvement as the framework for this quality improvement initiative,^21^ a key driver diagram was constructed to enhance postvisit communication from providers to caregivers in the institution’s multidisciplinary pediatric neuromuscular program (Fig. 1). The institutional review board at the Icahn School of Medicine at Mount Sinai approved the approach to consenting families for qualitative feedback and research. All patients or their caregivers provided their informed consent.

The initial steps in improving postvisit communication with the patients were to increase AVS provision, detailed in the intervention below, and evaluate the usefulness of those AVS by patients and families. Feedback from families and providers informed the next specific aim, addressing the medical jargon used in the patient instructions.

## INTERVENTIONS

### AVS Completion

**Fig. 2.**
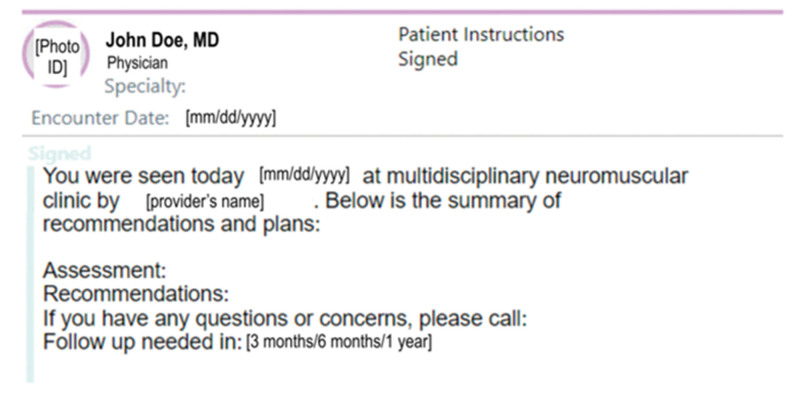
AVS patient instructions template used by providers.

The project’s initial phase focused on educating providers about the significance of the “patient instructions” portion of the AVS to achieve consistent completion following each encounter. An encounter was a clinical visit between a patient and a single provider. Each encounter produces one AVS. The specific aim during this phase was to increase the number of encounters with completed AVS patient instructions section to 80% over 6 months. Plan-Do-Study-Act cycles included (1) creation and implementation of a template text shortcut into the electronic medical record (EMR) for standardized patient instructions information; (2) regular review of critical elements with providers during a team huddle before each clinic session; (3) individualized feedback to clinicians regarding their completion rates. The text shortcut patient instructions template incorporated elements from the Family Experiences with Coordination of Care (FECC) surveys^22^ (a validated measurement tool of care coordination for children with medical complexity). The text shortcut included the name of the provider seen, an assessment section, a plan section, instructions on when the next appointment should be held, and who to contact for questions (Fig. 2). Given the importance of language-appropriate instructions,^19^ providers were regularly educated on where to access and use it. Because most patients seen in the clinic are either English or Spanish speakers, providers were reminded to translate their patient instructions into the appropriate language.

The project team then tracked the patient instructions completion rate from January 2021 until June 2023. The first intervention was in July 2021. After each clinic date, the project team evaluated the completion of AVS patient instructions through the EMR. An AVS patient instructions section was considered complete once the provider discussed the issue, filled out and signed the “Patient Instructions” note, and electronically released it to be printed or accessed via the patient portal. The process measure of completion percentages was obtained by dividing the total number of encounters with a completed patient instructions section by the total number of encounters after each clinic session. Then, a percentage completed for each specialty was generated to provide feedback before the next clinic session. The balancing measure was time to complete the AVS estimated by providers. Data were reviewed using control chart rules to identify special cause variation.^23^ Charts were created using QI Charts 2.0 software (version 2.0; Process Improvement Products, Austin, Tex.).

The project team also sought to obtain feedback from families attending the clinic to assess their AVS experience as a way of postvisit communication with providers, identify improvement areas, and inform next steps. The survey included a subset of messaging and care plan indicators from the FECC surveys^22^ to caregivers who consented during the clinic. Surveys were conducted by members of our project team in English or Spanish and were completed by caregivers online or via phone. Using phone and online questionnaires allowed for maximization of response rate and better representation of minority populations, as a previous study suggested.^21^ The questions surveyed caregivers on their satisfaction with the AVS as a means of postvisit communication. Specifically, caregivers were asked whether or not they found the AVS useful for the patient’s care, if it was easy to find, and whether or not the AVS content was easy to understand and included enough information. Additionally, the project team conducted open-ended interviews with available caregivers to obtain more specific feedback.

### Addressing Medical Jargon

The team focused on reducing medical jargon in the AVS patient instructions in response to family feedback and stakeholder review of providers’ documentation. This intervention aimed to reduce the number of medical jargon terms in the patient instructions by 25% over 3 months. To guide this effort, the project team created a presentation for providers outlining the classification of seven different types of jargon described by Pitt et al.^24^ Subsequently, each provider received personalized, written, specialty-specific feedback from the project team. This feedback included the percentage of patient instructions completed in the AVS per session and the mean number of jargon terms in patient instructions from the previous clinic date. Additionally, examples of suggested jargon-free replacement words were provided in the feedback (Table 1). The feedback was updated after each clinic session, sent out before the next session, and reviewed during team huddles before the session started.

**Table 1.** Sample Medical Jargon Education Sheet Given to Providers with Classified Areas of Medical Jargon with Suggested Replacements

Type of Jargon	Definition	Instead of...	Use...
Technical terminology	Words we didn’t know before our medical training	Scoliosis, spastic, cariogenic, nebulizer	Curved spine, tight muscle, promotes cavities, asthma machine *or define terms before using them*)
Alphabet soup	Acronyms and abbreviations	yo, s/p, h/o, MRI	Years old, following from, history of, brain scan *(or spell the abbreviations out*)
Medical vernacular	Familiar word but not understood	Ultrasound, tibia, gross motor skills, dislocation	body scan, leg bone, large movements, separated bones (*or define your terms*)
Medicalized English	Word with different meaning in the medical setting	Tolerance, appreciate, severe, global	Does well on, we can see that..., very, everywhere
Unnecessary synonyms	Exchanging known word for an unknown/more complicated one	Nonambulatory, bilateral, effective, Preventing, monitor	Cannot walk, on both sides, does well with, put a stop to, watch for
Euphemism	Attempts to soften the blow but making it more complicated	Expired, voided	Died, urinated
Judgmental jargon	Could be seen as derogatory despite no intent	Poor, failure	Not present, not strong enough, unable to do...

The mean and standard deviation of caregiver’s responses to relevant FECC questions are shown here. (N=21).

Each unique jargon term equaled a value of 1. Repeated jargon terms within the same note were not counted to facilitate the classification of unique jargon terms into a category. Additionally, counting repeated use of the same term would skew the data toward certain classification types. Jargon terms defined within the patient instructions were not counted. Two coders coded three clinic dates separately and compared scoring to ensure consistency. The project team met to discuss discrepancies and agree on a classification to be pursued with the rest of the coding. This was a continuous dialogue between the coder and the team. The same person then scored all remaining clinic dates. The project team calculated the outcome measure of total jargon use by adding the number of terms detected at each encounter.

## RESULTS

### AVS Completion

**Fig. 3.**
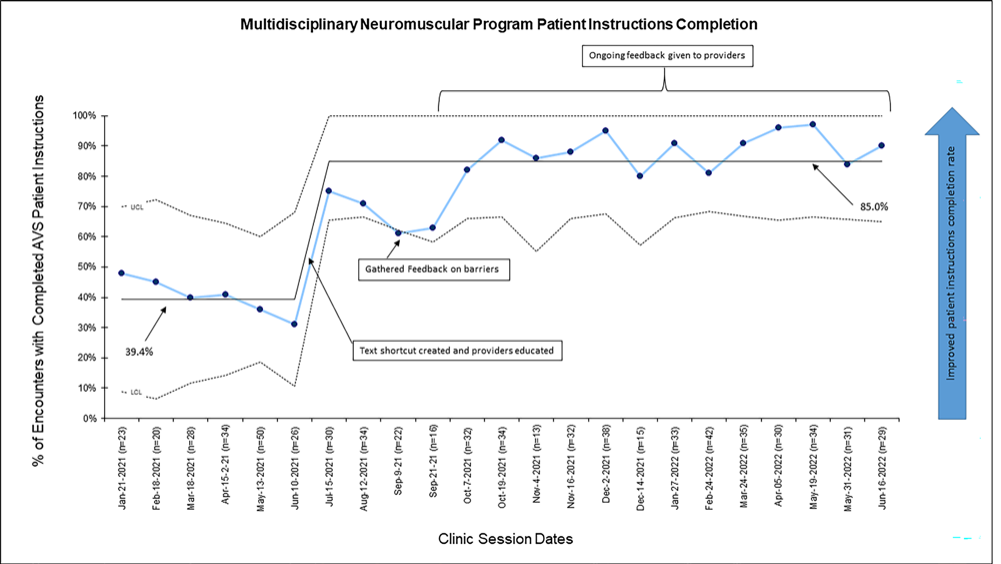
A control chart depicts improved AVS patient instructions completion rate per clinical session over time.

Baseline AVS completion rates for the six months before intervention demonstrated a mean of 39.4%. After education with providers, the completion rate of AVS patient instructions increased to a mean of 85.0% over 12 months (Fig. 3). Data continued to be intermittently collected to track sustainability, and, notably, there was a decrease in AVS completion rates at the beginning of the academic year when new trainees and providers joined the team. Re-education and reinforcement helped recover and sustain the completion rate, especially in the phase targeting medical jargon use. For the balancing measure, providers did not perceive that including the text shortcut in their patient instructions disrupted their clinical workflow.

### Feedback from Families

Twenty-one caregivers completed a portion of the FECC survey (Table 2) concurrently with the study team tracking the completion rate; 21 of the 53 (39.6%) caregivers who had consented answered the FECC survey. Most respondents (67%) reported that the AVS included the names of all the specialists caring for their child; 77% reported that it included information on what to do if issues arose after the visit, and 86% of the caregivers reported that the AVS was easy to understand. In-depth interviews with four caregivers reaffirmed the AVS’s usefulness but highlighted a need to make the AVS more concise and understandable. This need was articulated by quotes such as “It’s a lot, I was feeling overwhelmed looking at all that,” “What does BID mean?” or “It makes a difference for somebody who isn’t a good reader.” This feedback prompted the team to focus on reducing medical jargon to improve patient instructions’ readability and communication between providers and patients after their visit.

**Table 2.** Responses from Caregivers in FECC Indicators regarding Messaging and Communication

Question		N Answers (%)		
How often did the AVS include...		Always	Sometimes	Never
	*The names of all the specialist doctors who help* *care for your child?*	14 (66.7%)	4 (19.1%)	3 (14.2%)
	*The plan for follow-up care for your child after* *the visit?*	19 (90.5%)	2 (9.5%)	0 (0.00%)
	*What to do if your child had a problem after* *the visit?*	16 (76.2%)	2 (9.5%)	3 (14.2%)
**In the last 12 months...**		**Always**	**Sometimes**	**Never**
	*How often was the AVS easy to understand?*	18 (85.7%)	1 (4.76%)	2 (9.5%)
	*How often was the AVS useful to you and your* *family?*	17 (80.9%)	3 (14.2%)	1 (4.76%)
**Shared Care Plan**		**Yes**		**No**
	*Has the main provider created a shared care* *plan for your child?*	18 (85.7%)		3 (14.2%)
	*Do you have a copy of your child’s shared* *care plan?*	16 (76.2%)		5 (23.8%)

### Medical Jargon

**Fig. 4.**
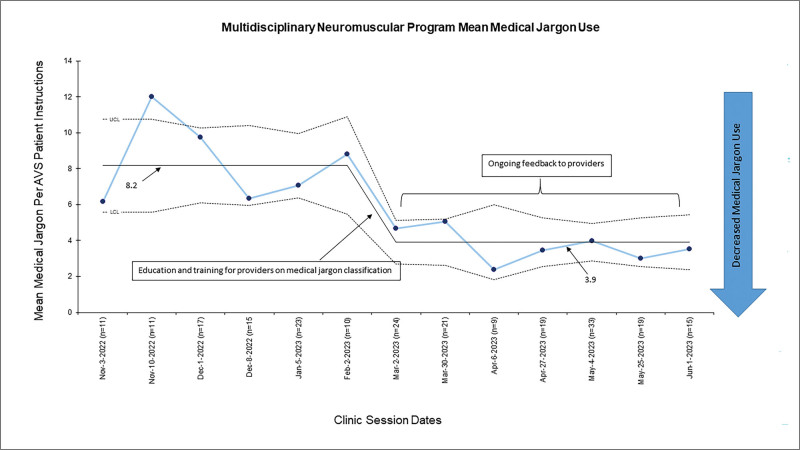
The control chart displays reduced medical jargon use per AVS patient instructions over time.

Baseline data of medical jargon was obtained from 87 preintervention AVSs for 26 patients seen over six clinic sessions. Intervention data were collected over 3 months, including 142 AVSs representing 30 children seen during one of seven clinic sessions. Total jargon decreased from a mean of 8.2 jargon terms per baseline AVS patient instructions to a mean of 3.9 jargon terms per AVS patient instructions (Fig. 4). This represents a 52.4% decrease in jargon per AVS patient instructions from baseline. The most common categories of jargon during both baseline and intervention periods were Alphabet Soup (33% of total jargon), Technical Terms (28% of total jargon), and Unnecessary Synonyms (24% of total jargon). All types of jargon were used except Euphemisms, which is usually a jargon used verbally.^24^ All specialties except one demonstrated a decrease in average jargon following the intervention, with one specialty exhibiting an 85.7% decrease (from 3.5 average jargon terms per encounter to 0.5 jargon terms per encounter) and another a 73.2% decrease (from 21.2 average jargon terms per encounter to 5.7 jargon terms per encounter). The specialty that did not exhibit a decrease in jargon was a specialty with a large provider turnover, contributing to less consistent provider-specific education.

## DISCUSSION

Multidisciplinary programs where children with medical complexity see multiple providers in one clinical session have rapidly grown. Yet, limited literature facilitates postvisit communication with families during these sessions.^1,3^ Using quality improvement methodology, the project team improved AVS patient instructions completion rate and reduced medical jargon in a pediatric multidisciplinary neuromuscular program for each clinical session over the targeted time. Education with providers, implementation of a template text shortcut, and frequent feedback contributed to successful change in this clinical setting. This quality improvement report is the first to evaluate written medical jargon in AVS. This project demonstrates there are tangible ways that multidisciplinary clinics can improve the delivery of important information to patients and families following a complex clinical visit.

Improving provider compliance and sustaining engagement was central to increasing the AVS patient instructions completion rate and reducing the prevalence of medical jargon. To do so, the project team used evidence from the literature to educate providers on the importance of the AVS^8,13,14,19^ and provided templates to illustrate expectations.^25,26^ Regular personalized feedback to each provider on the morning of clinic helped reinforce the importance of clear postvisit communication. Previous studies indicated that standardized written information increased caregiver understanding and compliance with postvisit directions.^15,27^ The EMR text shortcut implemented was structured to encourage critical communication elements from the validated FECC survey, such as providers’ contact information and plans for follow-up. Barriers to implementation included providers remembering the specific text shortcut and project team members being present during morning huddles to provide reminders. As noted by Williamson et al., healthcare workers are more invested in interventions when provided with real-time feedback and a way to appreciate their progress visually.^16^ Periods of decreased completion rates corresponded with times when feedback was not being provided to clinicians, highlighting the pivotal role of education and reinforcement in sustaining these goals. Functionally, the EMR did not consolidate the AVS of several providers for one patient into one composite document. As a result, multiple AVS documents were given to caregivers after a clinic session, which had the potential to overwhelm families and create redundancy for patients. The future state of the clinic would include having a coordinator concatenate all AVS for each patient and ensure no conflicting messages between providers. EMR systems would ideally be able to consolidate multiple AVS from one session to be provided to families.

Incorporating caregiver feedback on their experience with postvisit communication from their provider was critical to inform areas of focus.^28^ The responses to the FECC survey provided an opportunity to gather feedback from the caregivers and identify areas of improvement. These responses allowed the project team to refocus on decreasing medical jargon in the AVS patient instructions and ensure clear and concise notes. However, there were challenges in consistently recruiting many families to complete the FECC survey. Despite attempts with different outreach modalities (e-mail, phone call, text) and patient incentives, achieving a robust response rate to measure improvement over time was difficult. Caregivers of children with complex medical needs are very engaged with the healthcare system, and the families in the clinic are often from resource-poor populations.^29^ Those competing interests limit caregivers’ time to answer surveys and provide feedback. The results also had a very positive baseline response, influencing the project team’s decision to stop using this survey modality to measure and improve. Instead, the team shifted toward asking families more open-ended questions for more specific and directed feedback. Future recommendations include using a mixed-methods approach in obtaining patient feedback, conducting in-person interviews, and ensuring caregivers understand the goals and function of the AVS.

Similar to another study conducted using the Pitt et al. classification system for medical jargon,^30^ our project identified technical terminology, alphabet soup, and unnecessary synonyms as categories of jargon most commonly used by providers. This often stemmed from providers copying previous notes and using common medical abbreviations. To decrease jargon, the project team emphasized the importance of using simple language and distilling the main recommendations into patient instructions. This re-centered the AVS patient instructions section as a communication tool meant for families to have main take-away points in a written format.^8,13^ Studies demonstrated that providers often desire to establish good communication with patients and agree that minimizing medical jargon is important. Still, they often underestimate the amount of jargon usage.^31,32^ The suggested next steps include the creation of written jargon education modules that can be easily distributed to providers and automating feedback to providers.

Finally, this project has several limitations. One was the lack of objective data on the balance of time to AVS completion upon implementing the text shortcut template. The time taken to sign and complete each provider’s encounter may be a better proxy of whether additional time was used to complete AVS. As stated above, there was also a suboptimal response rate from families, limiting the project team’s understanding of the barriers families face when interacting with the AVS. Administering surveys on the phone may also have led to response bias,^33^ contributing to the overtly positive FECC answers. To minimize this bias, nonclinical members of the project team surveyed families. FECC answers collected did not have any comparison to a baseline point, and these answers were used more as a way to define the next directions in this quality improvement project. In addition, members of the project team conducted the jargon coding analyses. They made subjective decisions regarding classifying certain terms as jargon, which could lead to some reporter bias. In the future, EMR-embedded artificial intelligence could lead to more standardized jargon identification, allowing for longer sustainability of changed behavior.

## CONCLUDING SUMMARY

This quality improvement project provides concrete tools to improve postvisit communication with families by standardizing AVS patient instructions content and reducing its written medical jargon in a multidisciplinary setting with medically complex pediatric patients. Reminding providers of the importance of the AVS and providing personalized feedback are key to optimizing written content distributed to families. Leveraging technology could streamline AVS standardization and automated provider feedback in the future, allowing for easier replication of this project in other settings. Future work should focus on better quantifying caregiver perceptions of the AVS and best communication practices during multidisciplinary clinical encounters.

## ACKNOWLEDGMENTS

The authors would like to acknowledge the cooperation of the nursing, social work, administrative and medical staff in the project’s hospital, as well as our patients and families, for their valuable time and feedback. We want to thank Katie Gray for assisting in the project and all of the clinic’s providers for their engagement throughout the project. We would also like to acknowledge the Academic Pediatric Association’s Quality Improvement Scholars Program for their support throughout the project.
